# Comprehensive analysis of necroptotic patterns and associated immune landscapes in individualized treatment of skin cutaneous melanoma

**DOI:** 10.1038/s41598-023-48374-0

**Published:** 2023-11-30

**Authors:** Bo Yang, Pan Xie, Hongyu Huai, Junpeng Li

**Affiliations:** 1 Department of Ophthalmology, Chengdu Aier Eye Hospital, Chengdu, Sichuan China; 2 Department of Plastic and Burns Surgery, National Key Clinical Construction Specialty, The Affiliated Hospital of Southwest Medical University, Luzhou, Sichuan China; 3 Key Laboratory of Medical Electrophysiology, Ministry of Education & Medical Electrophysiological Key Laboratory of Sichuan Province, Institute of Cardiovascular Research, Southwest Medical University, Luzhou, Sichuan China

**Keywords:** Cancer, Biomarkers, Medical research, Oncology

## Abstract

Skin cutaneous melanoma (SKCM) constitutes a malignant cutaneous neoplasm characterized by an exceedingly unfavorable prognosis. Over the past years, necroptosis, a manifestation of inflammatory programmed cell demise, has gained substantial traction in its application. However, a conclusive correlation between the expression of necroptosis-related genes (NRGs) and SKCM patient's prognosis remains elusive. In this endeavor, we have undertaken an integrative analysis of genomic data, aiming to provide an exhaustive evaluation of the intricate interplay between melanoma necroptosis and immune-infiltration nuances within the tumor microenvironment. Through meticulous scrutiny, we have endeavored to discern the prognostic potency harbored by individual necroptosis-associated genes. Our efforts culminated in the establishment of a risk stratification framework, allowing for the appraisal of necroptosis irregularities within each afflicted cutaneous melanoma patient. Notably, those SKCM patients classified within the low-risk cohort exhibited a markedly elevated survival quotient, in stark contrast to their high-risk counterparts (*p* < 0.001). Remarkably, the low-risk cohort not only displayed a more favorable survival rate but also exhibited an enhanced responsiveness to immunotherapeutic interventions, relative to their high-risk counterparts. The outcomes of this investigation proffer insights into a conceivable mechanistic underpinning linking necroptosis-related attributes to the intricacies of the tumor microenvironment. This prompts a conjecture regarding the plausible association between necroptosis characteristics and the broader tumor microenvironmental milieu. However, it is imperative to emphasize that the pursuit of discerning whether the expression profiles of NRG genes can indeed be regarded as viable therapeutic targets necessitates further comprehensive exploration and scrutiny. In conclusion, our study sheds light on the intricate interrelationship between necroptosis-related factors and the tumor microenvironment, potentially opening avenues for therapeutic interventions. However, the prospect of translating these findings into clinical applications mandates rigorous investigation.

## Introduction

Dysfunctions within the neuroendocrine and immune systems localized in the skin, the largest organ in the human body, can precipitate various disorders, including melanoma^1^.Cutaneous melanoma represents a malignant neoplasm originating from melanocytes and stands as the predominant subtype within the spectrum of melanoma. Despite accounting for less than 5% of all cutaneous malignancies, it emerges as the foremost contributor to global skin cancer-related fatalities, assuming the position of the most lethal form of skin cancer^2,3^. In the backdrop of an escalating mortality trend attributed to malignant melanoma in recent times, the prognostic outlook for patient survival exhibits notable heterogeneity contingent upon distinct melanoma classifications. Specifically, the 5-year survival rate for individuals afflicted with stage 0 cutaneous melanoma ascends to 97%, a stark juxtaposition to the mere 10% survival rate characterizing patients grappling with stage IV cutaneous melanoma^4^. While early-stage cutaneous melanoma can be effectively addressed through wide-ranging excision, the majority of diagnoses manifest at advanced stages, underscoring the urgency of early detection and intervention^5^. Notwithstanding the current decline in melanoma-associated mortality attributed to modalities encompassing immune checkpoint therapy, targeted therapeutic approaches, radiotherapy, and chemotherapy^6^, notably in the context of the B-Raf proto-oncogene serine/threonine kinase (BRAF) V600 (Val600) mutation, the application of select BRAF inhibitors in conjunction with schizogen-activated protein kinase inhibitors has evinced a substantial enhancement in treatment response and overall survival rates^7^. However, the pervasiveness of primary or acquired resistance translates into a significant cohort of melanoma patients experiencing unresponsiveness or relapse in the face of anti-PD1 and CTLA-4 therapeutic regimens. This phenomenon not only signifies immunotherapeutic setbacks but also underscores the pressing imperative to ascertain more efficacious treatment modalities^8,9^. Hence, the pursuit of novel prognostic biomarkers assumes paramount importance in the quest to elucidate effective therapeutic paradigms, consequently ameliorating the overall prognosis for individuals contending with cutaneous melanoma.

Apoptosis resistance stands as a significant contributor to the ineffectiveness of chemotherapy in cancer treatment. In instances where intracellular apoptotic signaling is lacking, an alternative non-apoptotic cell demise pathway, known as necroptosis, emerges as a potential activation route ^10^. Necroptosis, characterized as a regulated variant of necrotic cell death, operates through a cysteine-independent process, primarily orchestrated by key mediators including receptor-interacting protein 1 (RIP1), RIP3, and mixed-spectrum kinase structural domain-like (MLKL). This mechanism not only bypasses apoptosis resistance but also holds the potential to incite and bolster antitumor immune responses within the ambit of cancer therapy^11^. Furthermore, the ramifications of necroptosis extend to its involvement in the etiology and progression of diverse immune-related disorders, spanning acute kidney injury, acute hepatitis, inflammatory skin conditions, inflammatory bowel disease, pathological oncogenic processes, and an array of systemic maladies^12,13^. Nonetheless, the intricate modus operandi through which necroptosis influences oncogenesis remains enigmatic. On one facet, necroptosis exerts the capacity to trigger robust adaptive immune reactions, effectively impeding tumor advancement. Conversely, the inflammatory milieu triggered by necroptosis might concurrently foster tumorigenesis and metastasis, potentially culminating in an immune-suppressive microenvironment conducive to tumor growth^11^. It is worth noting, however, that the mechanistic underpinnings of necroptosis within the context of melanoma remain relatively unexplored, with only a limited number of investigations delving into this facet^14,15^.

In the present investigation, we commenced by extracting expression profiles about genes associated with necroptosis from the TCGA-SKCM dataset. Through a systematic analysis, we discerned independent prognostic determinants intricately linked to patient outcomes, subsequently culminating in the establishment of prognostic indicators rooted in necroptosis in melanoma. This was achieved through rigorous prognostic assessment. Augmenting our endeavor, an exploration of the immune microenvironment was undertaken to unravel the underlying immunological mechanisms at play in melanoma. Notably, this endeavor served to corroborate a robust and substantive correlation existing between the derived risk score and the immune microenvironment. By leveraging these findings, the population of melanoma patients can be effectively stratified into discrete high-risk and low-risk clusters, predicated on distinctive molecular signatures. Employing a risk-stratified approach, a survival analysis was conducted, thereby affording an evaluative lens to scrutinize the prognostic landscape for individuals afflicted by melanoma. In amalgamation, the overarching objective of this study resides in the conception of an innovative prognostic prediction model, one grounded in the genomic underpinnings of necroptosis-associated genes. Through its implementation, we envision the provision of a valuable clinical framework, one that lends itself as a diagnostic and therapeutic reference tool in the context of melanoma management.

## Materials and methods

**Figure 1:**
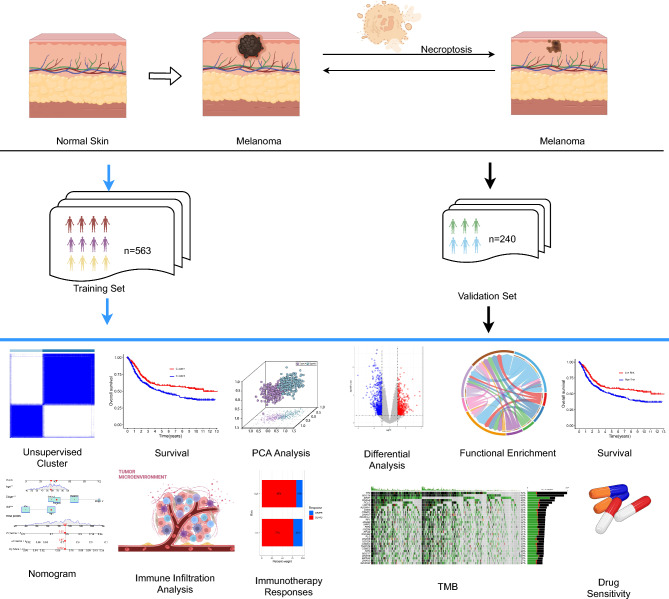
Flow chart for comprehensive analysis of necroptosis patterns in SKCM patients.

The transcriptomic data for TCGA-SKCM, represented by FPKM values, along with clinically pertinent information, were procured from the UCSC website (https://xenabrowser.net/datapages/). To mitigate the impact of confounding variables, patients characterized by incomplete clinical records and an overall survival duration of less than 30 days were excluded from the study cohort. Melanoma samples from GSE65904, GSE53118, GSE54467, GSE19234, and GSE15605 were sourced from the Gene Expression Omnibus (GEO) platform (https://www.ncbi.nlm.nih.gov/geo/) in their raw CEL and TAR file formats^16–20^. The GSE19234 and GSE15605 microarray datasets underwent background correction and normalization through the employment of the RMA function within the "Affy" package^21^. As for GSE53118, GSE54467, and GSE65904, the raw data was subjected to background correction and normalization using the lumiExpresso function within the R package "lumi"^22^. The RNA-seq data originating from TCGA-SKCM underwent conversion into TPM values. The amalgamation of TCGA data from the four GEO cohorts, alongside the rectification of batch effects, was executed employing the ComBat function from the "sva" R package^23^. Necroptosis-associated genes were systematically retrieved from GeneCards (https://www.genecards.org/), considering only those exhibiting correlation coefficients surpassing 1^24^. This meticulous curation culminated in the inclusion of a total of 941 genes for subsequent analyses (Supplementary Table S1). All ensuing analyses were performed using R (version 4.2.2) in conjunction with the suite of R Bioconductor packages. The flow chart of this study is shown in Fig. 1.

### Unsupervised cluster analysis of NRGs

Patients were systematically categorized into distinct molecular subgroups predicated on the expression patterns of NRG through the utilization of an unsupervised clustering methodology, facilitated by the R package "ConensusClusterplus"^25^. The generated clusters were subjected to a cumulative distribution function (CDF) analysis, which exhibited a seamless increment, indicative of ample representation within each subtype, bolstered by a substantial sample size. To render the findings accessible, both heatmap and Kaplan–Meier survival curves were employed as visualization tools. In an endeavor to unravel nuanced distinctions, a comparative assessment was undertaken concerning the expression profiles of major histocompatibility complexes and T-cell stimulatory factors across the delineated clusters.

### Functional enrichment analysis

Enrichment analyses were conducted utilizing Gene Ontology (GO) and Kyoto Encyclopedia of Genes and Genomes (KEGG) databases^26,27^. The "clusterProfiler" R package was instrumental in executing these analyses, and the outcomes were effectively rendered visually through the utilization of the "ggplot2" package^28,29^. To further elucidate the genomic variations, the Gene Set Variation Analysis (GSVA) method was employed, with c2.cp.kegg.Hs.symbols.gmt obtained from the MSigDB database being utilized as the definitive gene set for this purpose^30,31^.

### Construction of a prognostic model for necroptosis

Differential gene expression analysis across distinct necroptosis subtypes was conducted employing the "limma" package, employing a defined threshold of |logFC|> 1.0, along with a stringent adjusted *p* value threshold of less than 0.05^32^. To elucidate the prognostic implications of these identified differential genes, a Cox regression analysis was undertaken. Subsequently, prognostic gene candidates were further refined through a least absolute shrinkage and selection operator (LASSO) analysis, employing the "glmnet" R package^33^. The optimal minimum λ value was determined during this process. Genes shortlisted for constructing risk models were ascertained via a multivariate Cox regression analysis. A random seed of 14 was set, enabling the random division of the dataset into training and test subsets at a 7:3 ratio. The training subset was then harnessed for the establishment of a risk prediction scoring model associated with necroptosis-related genes. Leveraging the median value of the risk score, patients within the training set were effectively categorized into distinct low-risk and high-risk strata. Subsequent comparison of the overall survival trends of these stratified groups was achieved through Kaplan–Meier analysis. To quantitatively evaluate the predictive performance of the model, receiver operating characteristic (ROC) curves were utilized in conjunction with the area under the curve (AUC) metric^34^. Validation of the developed risk scoring model was executed utilizing the independent test dataset. Last but not least, ROC curves were used to compare the developed models to those of Gang Hu et al. and Zehao Niu et al., and to assess how well the three models predicted outcomes^35,36^.

### Creation of nomogram

Clinical attributes of SKCM patients were acquired and subsequently integrated with the developed genetic prognostic model. Employing the R package "rms"^37^, a multifactorial Cox regression analysis was undertaken to craft a comprehensive nomogram model, encompassing joint prognostication. Furthermore, this process facilitated the creation of a visual nomogram representation. In a bid to rigorously evaluate the predictive potential of the nomogram model, a suite of analytical tools was engaged. The time-dependent receiver operating characteristic (TimeROC) analysis^38^, calibration curves, and decision curve analysis (DCA) curves were systematically employed^39^. These evaluative mechanisms collectively gauged the model's precision in predicting outcomes and its clinical utility.

### Construction of a diagnostic model

We created a diagnostic model based on these two genes using logistic regression and validated it in the GSE15605 dataset in order to further assess the diagnostic effectiveness of the important risk genes DLL3 and SEMA6A^40^.

### Tumor microenvironment and immune checkpoint assessment

The immune infiltration score was evaluated using the "ESTIMATE" algorithm^41^. For a comprehensive assessment of immune cell infiltration across distinct samples, the "CIBERSORT" package was employed, facilitating the calculation of abundances for 22 distinct infiltrating immune cell types. Employing the "CIBERSORT" algorithm^42^, we conducted a comprehensive analysis of the relative abundance of 22 human immune cell subpopulations within SKCM samples. Furthermore, a focused inquiry into immune regulatory mechanisms was undertaken through the assessment of 33 immune checkpoints (Supplementary Table S2). This analysis sought to uncover potential variations in expression profiles across distinct risk groups.

### Mutation and drug sensitivity analysis

The investigation into melanoma mutations was conducted through the utilization of the "MAFTOOLS" software tool^43^. Additionally, an in-depth analysis of drug sensitivity was carried out, employing the "oncoPredict" package^44^. This analysis aimed to ascertain the half maximal inhibitory concentration (IC50 values) of frequently utilized chemotherapeutic agents in the context of melanoma treatment.

### Cell culture and real-time quantitative polymerase chain reaction

From the Chinese Academy of Sciences Cell Bank, the normal human fibroblast cell line HF and the human melanoma tumor cell line A375 were acquired. All cells were cultured in Dulbecco's modified Eagle's medium (DMEM) supplemented with 10% fetal bovine serum (Gibco, Thermo Fisher Scientific, Inc.) at 37 °C in an incubator with 5% carbon dioxide. Cell cultures were established in 10 cm dishes with an initial inoculum density of 80 × 10^4^ cells and were subsequently maintained at 37 °C. Cells were harvested once they reached 80% confluence. The Molpure® Total Cell/Tissue RNA Kit (produced by YEASEN) was used to initially harvest the cells from the 10 cm dishes and extract the RNA. Reverse transcription PCR (RT-PCR) was used to amplify cDNA after that. The primers mentioned in Supplementary Table 8 were used to perform real-time quantitative PCR (qPCR), which was the final step in quantifying mRNA. For the qPCR procedure, the QuantStudio TM3 machine from ThermoFisher was used with the PrimeScript RT kit from PhDL Biotech. For qPCR temperature changes, 35 s at 95 degrees, 30 s at 55 degrees, and 45 s at 72 degrees were used. Using a QuantStudio TM3 machine from ThermoFisher, the qPCR was carried out. The 2^−ΔΔCT^ approach was used to determine how much the target genes' expression had changed.

### Statistical analysis

Statistical analyses were carried out using R (version 4.2.2). To quantify the strength and direction of associations, Spearman correlation analyses were conducted to compute correlation coefficients. For the comparison of multiple group variables, the chi-square test was employed. The assessment of overall survival between patients categorized into high-risk and low-risk groups was accomplished using the log-rank test within the Kaplan–Meier analysis framework. In alignment with convention, a statistically significant distinction was defined by a *P* value of 0.05 or lower.

### Ethics approval and consent to participate

This is an observational study. The Research Ethics Committee of Southwest Medical University Hospital has confirmed that no ethical approval is required.

## Result

### Identification of the SKCM necroptosis cluster

**Figure 2:**
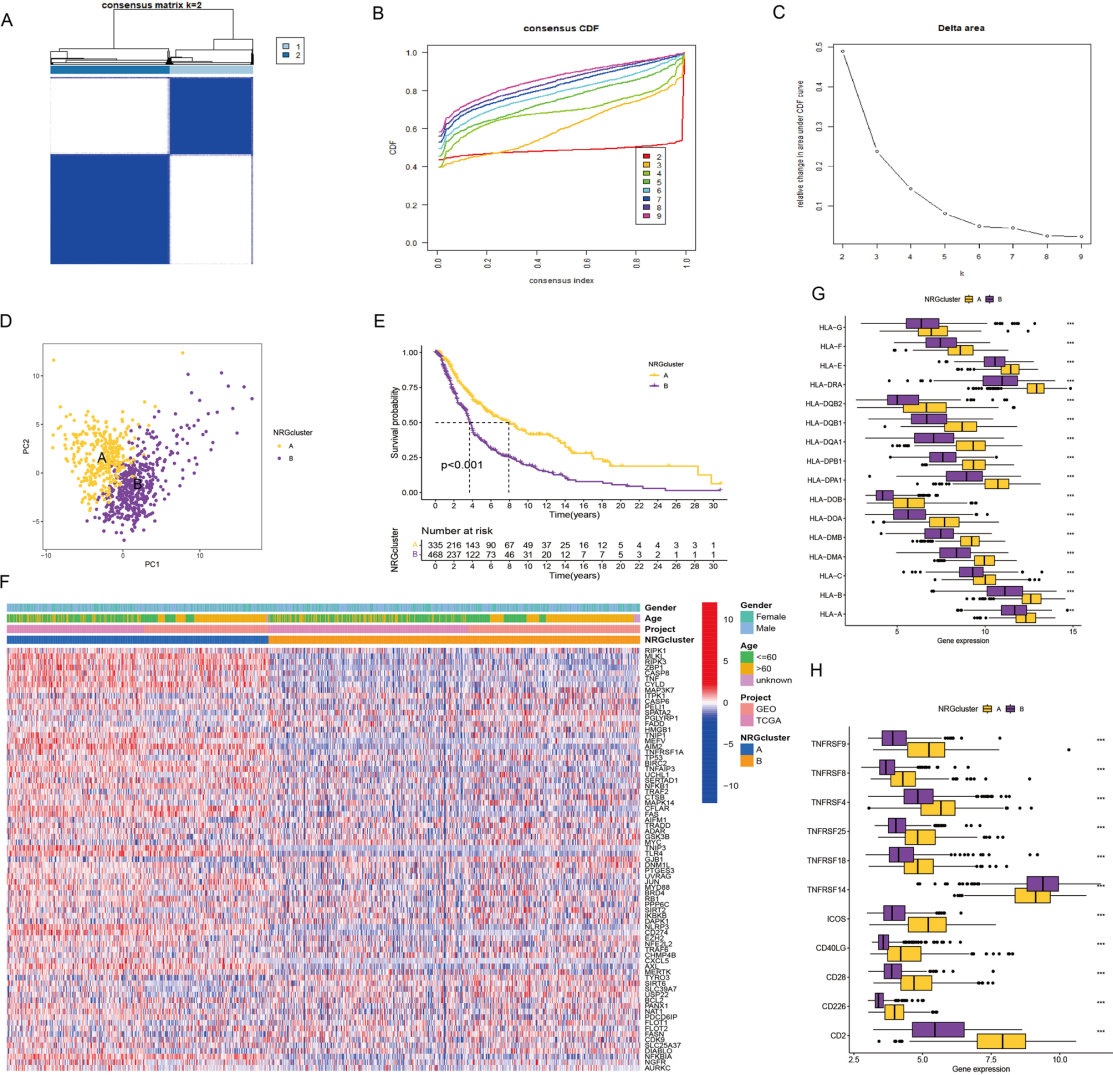
(**A**–**C**) Define a heatmap of the consistency matrix for two clusters (k = 2) and their associated regions. (**D**) PCA results showed that these two clusters differed significantly in transcriptional expression. (**E**) Survival analysis curves of SKCM patients with two NRG modification patterns. (**F**) Unsupervised clustering of necroptosis genes in SKCM cohorts. (**G**–**H**) Gene expression of HLA and MHC gene sets between two distinct clusters. Statistical significance at the level of * < 0.05, ** < 0.01, and *** < 0.001.

The comprehensive analysis encompassed a total of 803 patients drawn from five distinct melanoma cohorts, namely, TCGA-SKCM, GSE65094, GSE53118, GSE54467, and GSE19234. Employing an unsupervised clustering algorithm, we stratified melanoma patients based on their NRG expression profiles. This facilitated a deeper exploration of the distinctive characterization of NRGs within the context of melanoma. Remarkably, the outcomes of the clustering analysis delineated two discernible groups, denoted as group A (n = 335) and group B (n = 468), when utilizing k = 2 (Fig. 2A,B,C). The validity of this clustering was substantiated through the employment of a heatmap, while principal component analysis (PCA) further accentuated the existence of these two distinctive clusters. Intriguingly, this clustering was also effectively captured by a PCA plot (Fig. 2D). Subsequent Kaplan–Meier survival analysis demonstrated that patients belonging to the SKCM subtype A exhibited markedly extended overall survival duration when compared with their counterparts in the subtype B group (Fig. 2E). Research into the interactions between these two clusters and different ages and genders showed that NRG cluster A expressed NRGs more than cluster B (Fig. 2F).

### Patients with different molecular subtypes exhibit different immune infiltration states

Immunological assays were systematically conducted to delve into the discernible variations in immune infiltration across distinct molecular subtypes. Through the implementation of GSVA enrichment analysis, notable statistically significant disparities between immune cell abundances were unveiled between cluster A and cluster B, as discerned from the ssGSEA outcomes (Supplementary Figure S1A; Supplementary Table S3)^45^. This discrepancy was particularly evident, except for the CD56^dim^ natural killer cell subset, wherein the subcluster A exhibited a more favorable immune infiltration abundance as opposed to subcluster B (Supplementary Figure S1B). Additionally, a comprehensive scrutiny of the interrelation between the two subtypes and major histocompatibility complexes, along with T-cell stimulating factors, was undertaken. The examination of this association demonstrated that the subpopulation A consistently displayed elevated expression levels of major histocompatibility complex components and T-cell stimulating factors, substantiating the inclination towards heightened immunomodulation in this subset. Notably, this trend held across several factors, with the exception being TNFRSF14 (Fig. 2G,H).

### DEGs-based analysis of necroptosis clusters

**Figure 3:**
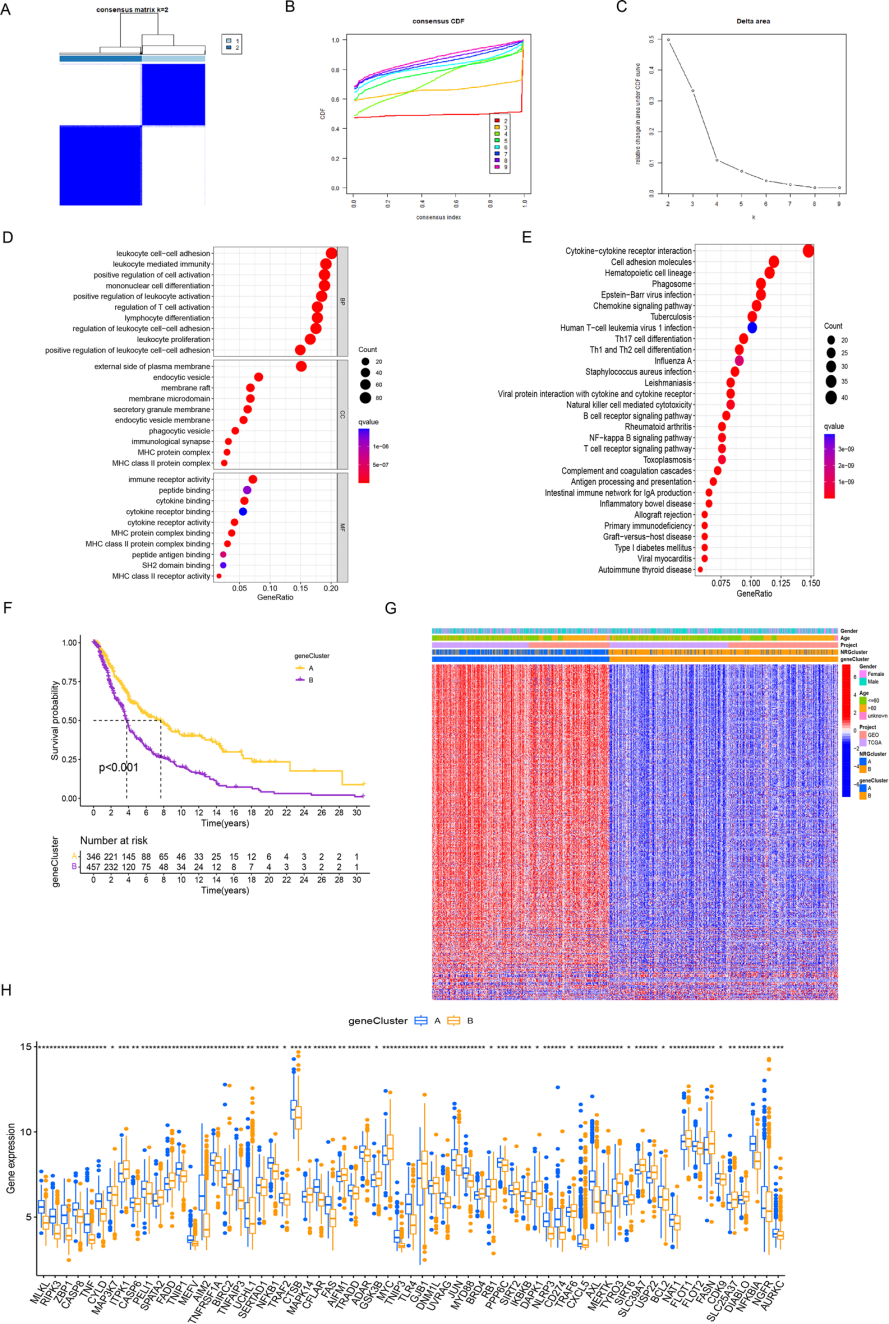
(**A**–**C**) Cluster analysis of DEGs identified two gene clusters. (**D**–**E**) GO and KEGG enrichment analysis of DEGs in two gene clusters. (**F**) Survival analysis of the two gene clusters. (**G**) Unsupervised clustering was used to study DEGs. Two gene clusters and clinical characteristics are connected. (**H**) Two gene clusters, 65 necroptosis genes, and their varying modes of expression.

Employing the "limma" R package, we successfully pinpointed 447 differentially expressed genes (DEGs) intricately linked to necroptotic clusters. Subsequently, prognostic gene candidates were discerned via univariate Cox regression analysis (Supplementary Table S5). This analytical trajectory gave rise to the identification of two distinct gene clusters, aptly denoted as gene clusters A and B (Fig. 3A,B,C). The classification of these clusters aligned closely with the degree of necroptosis, thus furnishing a substantial delineation. Functional insights into the roles of the DEGs were gleaned through a robust enrichment analysis that encompassed the GO and KEGG databases. The GO analysis prominently underscored that the majority of the identified differential genes were intricately associated with biological processes such as leukocyte cell–cell adhesion, leukocyte-mediated immunity, and positive regulation of cell activation. At the cellular level, the enriched components primarily revolved around the external side of the plasma membrane, endocytic vesicles, and membrane rafts. Moreover, molecular functions were significantly enriched for attributes encompassing immune receptor activity, peptide binding, and cytokine binding. Intriguingly, the KEGG pathway analysis underscored the engagement of DEGs in pivotal biological pathways, including cytokine-cytokine receptor interaction, cell adhesion molecules, and hematopoietic cell lineage (Fig. 3D,E; Supplementary Table S4). The clinical ramifications of these findings were evident through Kaplan–Meier survival analysis, which distinctly demonstrated that patients grouped within cluster B faced markedly inferior overall survival when juxtaposed with their counterparts in cluster A (Fig. 3F). The gene expression data revealed significant differences in the NRG expression patterns between the two gene clusters (Fig. 3G,H).

### Risk scoring model for necroptosis gene correlation

**Figure 4:**
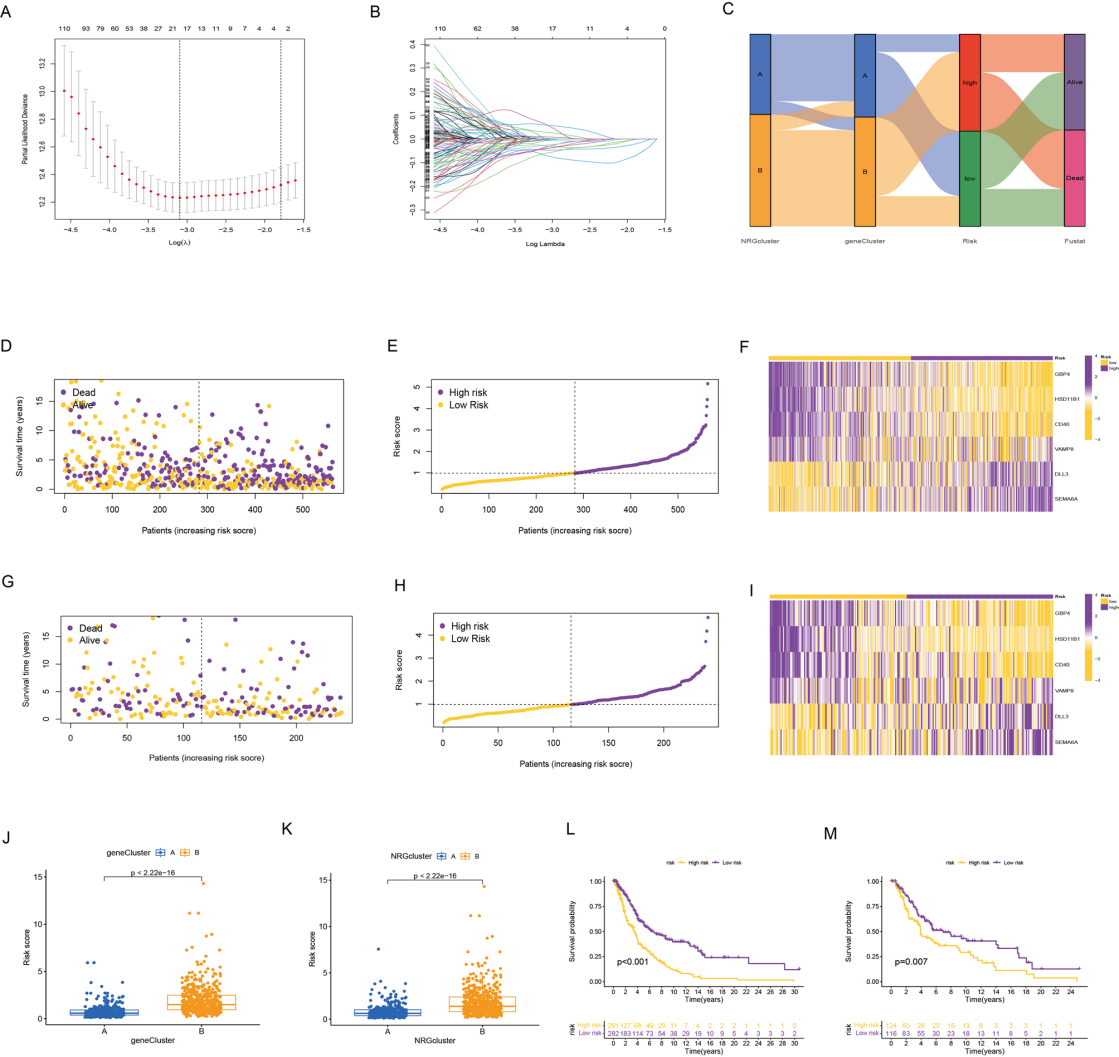
(**A**–**B**) LASSO regression identifies optimal risk genes. (**C**) Sankey plot showing changes in NRG clusters, gene clusters, and status. (**D**–**I**) In the training and test groups, Necroptosis risk score distribution and patient survival status are plotted in ranked point and scatter plots. Genes associated with risk expression and distribution. (**J**) Differences in necroptosis risk scores in two gene clusters. (**K**) Differences in necroptosis risk scores in two NRG clusters. (**L**–**M**) Survival analysis for patients with high or low necroptosis risk scores in the training and test groups.

A necroptosis-associated risk score model was meticulously constructed, hinging on the differential genes affiliated with distinct gene clusters. Utilizing LASSO regression, the optimal λ-value was derived, leading to the identification of 18 risk genes (Fig. 4A,B). Subsequently, this pool of 18 risk genes was subjected to multivariate Cox regression analysis, ultimately yielding a refined set of 6 genes (GBP4, HSD11B1, CD40, VAMP8, DLL3, SEMA6A). The necroptosis risk model was meticulously formulated as follows, yielding the risk score equation: GBP4 exp *(− 0.1395) + HSD11B1 exp *(− 0.1775) + CD40 exp *(− 0.1483) + VAMP8 exp * 0.1994 + DLL3 exp * 0.0842 + SEMA6A exp * 0.1122. The clinicopathologic features associated with the high and low risk groups are shown in Supplementary Table S7. Sankey plot provided an insightful visualization of the intricate relationships among patients across two distinct NRG clusters, two gene clusters, as well as high- and low-risk groups (Fig. 4C). Notably, the risk-scoring formulas rooted in the risk model effectively stratified the training set into high- and low-risk groups, resulting in divergent survival outcomes (Fig. 4D,E,F,G,H,I). The risk distribution plots of the necroptosis risk model corroborated a consistent trend: escalating necroptosis risk scores paralleled heightened mortality risk and correspondingly diminished survival duration. This correlation was succinctly captured in the scatter plot, where a preponderance of high-risk patients exhibited truncated survival times. Furthermore, the model analysis of the necroptosis risk score concerning gene expression patterns underscored VAMP8, DLL3, and SEMA6A as high-risk genes, while GBP4, HSD11B1, and CD40 assumed the mantle of low-risk genes. Gene cluster A was linked to lower risk ratings, whereas gene cluster B was linked to higher risk scores. In NRG clustering, cluster A was linked to a decreased necroptosis risk score. Higher risk ratings and NRG cluster B were significantly correlated (Fig. 4J,K). Subsequent Kaplan–Meier survival analysis for both training and test cohorts accentuated that the high-risk group suffered a higher incidence of mortality relative to the low-risk cohort. This disparity was further substantiated by the statistically significant differences observed within Kaplan–Meier survival curves (Fig. 4L,M). To validate these findings, an entirely new test group was incorporated, affirming the robust association between necroptosis risk scores and patient survival. Elevated necroptosis risk scores were inextricably linked to elevated mortality risk and concomitantly abbreviated survival span. This was graphically exemplified by the Kaplan–Meier survival curve, where patients boasting high necroptosis scores exhibited markedly lower survival rates compared to those with lower scores (Supplementary Figure S1C,D,E,F). The ROC curve indicates that our model has an AUC value of 0.744, which is much higher than the AUC values for the other two necroptosis-related models (Supplementary Figure S2B).

### Diagnostic modeling of key risk genes

Using data from the GSE15605 dataset, we verified the diagnostic model built for DLL3 and SEMA6A's predictive effectiveness. ROC curves for the above two genes were used to examine how well individual genes could distinguish between melanoma and normal samples. All of the genes' AUCs were higher than 0.7, and the logistic regression model's AUC was 0.857 (95% CI: 0.766–0.92), indicating that it was more accurate and specific than the individual marker genes for melanoma and normal sample differentiation (Supplementary Figure S2C,D). We investigated the expression patterns of the aforementioned two genes and their association with prognosis utilizing the GEPIA online database. Our analysis revealed that both genes exhibited significantly elevated expression levels in SKCM in comparison to normal tissues (Supplementary Figure S2F,G). Additionally, it was observed that patients characterized by lower expression levels of DLL3 and SEMA6A exhibited a prolonged OS period (Supplementary Figure S2J,K). Ultimately, the expression profiles of these two genes were corroborated through experimentation with cell lines, yielding results consistent with those obtained from the database analysis (Supplementary Figure S2H,I).

### Tumor microenvironment and immunotherapy

According to the immunoassay results obtained through "CIBERSORT", the six necroptosis risk model genes exhibited a substantial association with various immune cell types (Supplementary Table S6). For instance, GBP4 demonstrated a positive correlation with immune cell populations like CD8^+^ T cells, activated CD4^+^ T memory cells, and M1 macrophages. In contrast, DLL3 exhibited a negative correlation with immune cells such as M2 macrophages and resting CD4^+^ T memory cells. The interplay between these genes and immune cells was robustly demonstrated. Furthermore, the necroptosis risk score exhibited intricate correlations with distinct immune cell subsets. Specifically, the risk score showcased a negative correlation with plasma cells, activated memory CD4^+^ T cells, M1 macrophages, and CD8^+^ T cells, while a positive correlation was noted with M0 macrophages (Supplementary Figure S3A, C).

**Figure 5:**
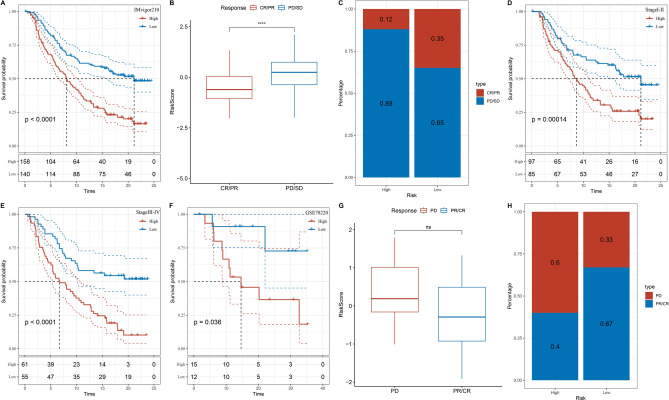
(**A**) Prognostic differences among risk score groups in the IMvigor210 cohort. (**B**) Differences in risk scores for immunotherapeutic response in the IMvigor210 Cohort. (**C**) Distribution of immunotherapy responses between risk scoring groups in the IMvigor210 Cohort. (**D**–**E**) Prognostic differences between risk score groups for early and advanced stage patients in the IMvigor210 Cohort. (**F**) Prognostic differences among risk score groups in the GSE78220 cohort. (**G**) Differences in risk scores for immunotherapeutic response in the GSE78220 cohort. (**H**) Distribution of immunotherapy responses between risk scoring groups in the GSE78220 cohort.

The comprehensive exploration of immune infiltration within high- and low-risk groups, as assessed through the "ESTIMATE" algorithm, yielded noteworthy findings. Immune score, stromal score, and ESTIMATE score were all significantly higher in the low-risk group compared with the high-risk group (Supplementary Figure S3B). This divergence implied that the low-risk group harbored a markedly greater abundance of immune infiltration. Furthermore, the connection between our risk model and immune checkpoints was systematically examined. Among the 33 immune checkpoints, including LAG3, IFNG, and CTLA-4, significant differential expression was observed across the two distinct subgroups (Supplementary Figure S2E). Most immune checkpoints exhibited a positive correlation with the low-risk group, indicating that individuals within this subgroup might be more responsive to immunotherapy (Supplementary Figure S3D). To substantiate the sensitivity of various risk subgroups to immunotherapy, external datasets encompassing patients who underwent anti-PD-L1 therapy (IMvigor210 and GSE78220) were leveraged as test cohorts^46,47^. Remarkably, patients demonstrating complete response (CR) and partial response (PR) exhibited significantly prolonged survival compared to patients with stable disease (SD) and progressive disease (PD). Notably, within the IMvigor210 cohort, an intriguing discrepancy was detected in the response subtypes (CR, PR, SD, and PD) across different necroptosis subtypes (*p* < 0.05). Higher-risk patients displayed a larger proportion of SD/PD, while CR/PR occurrences were more prevalent in the lower-risk group. Although no substantial variation in response subtypes to treatment-free therapy was evident between high- and low-risk patients within the GSE78220 cohort, the profound impact of risk stratification was consistently observed in the context of patients receiving immunotherapy (Fig. 5A,B,C,D,E,F,G,H). Through the establishment of risk-based subgroups within the GSE78220 and IMvigor210 cohorts, employing an optimal cutoff value derived from the "survivalROC" R package^48^, significant differences in overall survival emerged. Patients within the low-risk group showcased considerably prolonged overall survival in both the IMvigor210 and GSE78220 cohorts, transcending the stages of the disease. These findings compellingly suggest that the low-risk subgroup exhibited a more favorable therapeutic response to immunotherapy compared with their high-risk counterparts. This underlying rationale elucidates the superior survival outcomes of melanoma patients within the low-risk group compared to those within the high-risk group.

### Mutation analysis and drug sensitivity

**Figure 6:**
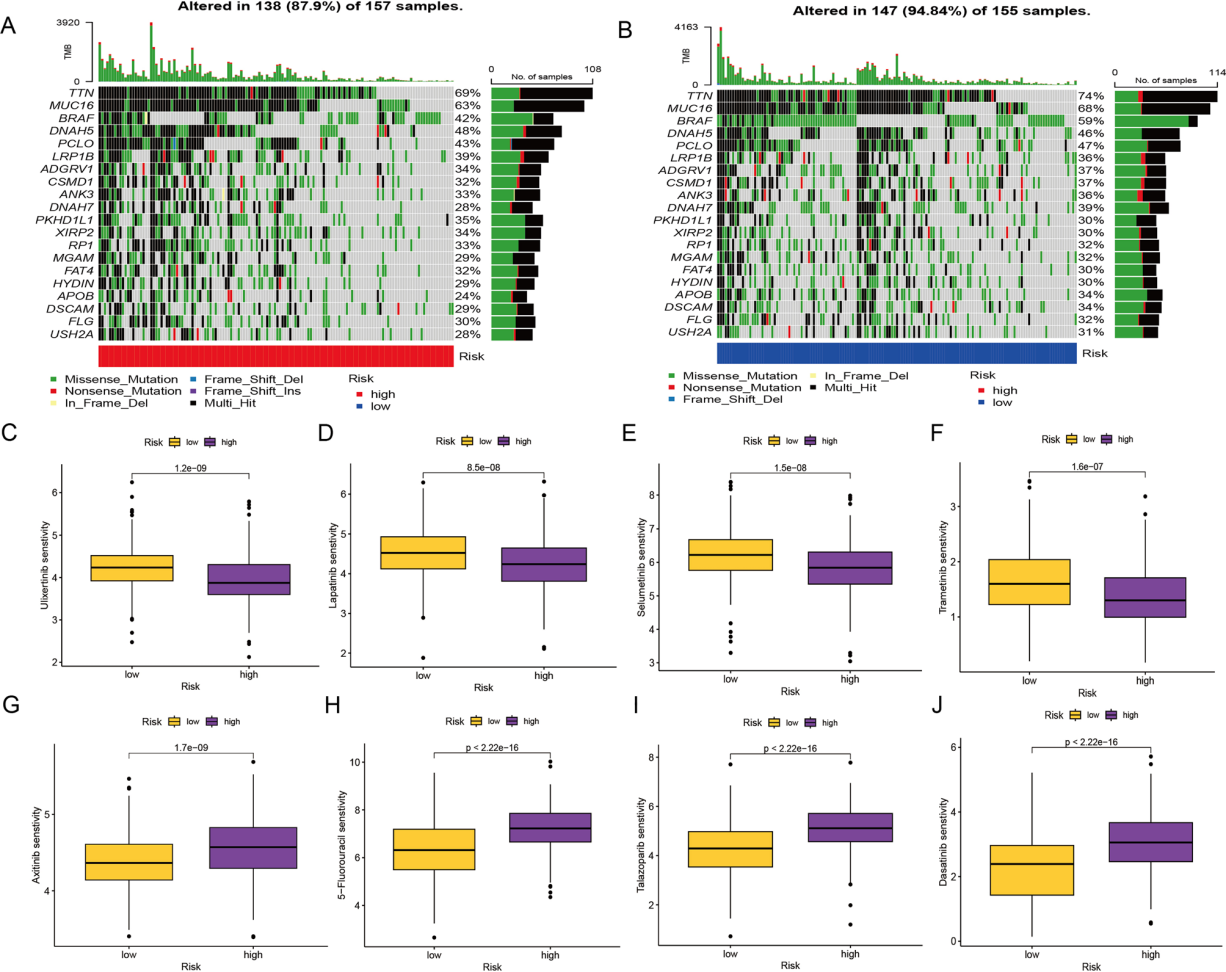
(**A**–**B**) A waterfall plot depicts the characterization of somatic mutations in the necroptosis risk model. (**C**–**J**) Necroptosis risk model-assisted selection of antitumor drug candidates.

The analysis of waterfall plots vividly illustrates that the tumor mutational burden (TMB) is notably elevated within the low-risk population as opposed to the high-risk counterpart. This discrepancy assumes significance given the well-documented impact of tumor mutation burden on prognosis. Indeed, patients with elevated TMB have been consistently shown to exhibit extended survival durations relative to those with diminished TMB levels (Fig. 6A,B). Furthermore, a meticulous inquiry into the sensitivity of distinct subpopulations to various chemotherapeutic agents holds potential significance in informing clinical interventions, particularly for patients within the high-risk group. Notably, patients belonging to the high-risk category manifest heightened sensitivity to a selection of drugs, including Lapatinib, Selumetinib, Trametinib, and Ulixertinib (Fig. 6C,D,E,F). On the other hand, individuals categorized within the low-risk group exhibit greater responsiveness to chemotherapeutic agents such as 5-Fluorouracil, Axitinib, Dasatinib, and Talazoparib (Fig. 6G,H,I,J). This intricate understanding of drug sensitivity across distinct risk groups serves as a crucial theoretical cornerstone for the tailored clinical management of patients, especially those situated within the high-risk subgroup.

### The risk score can be an independent prognostic factor for SKCM

**Figure 7:**
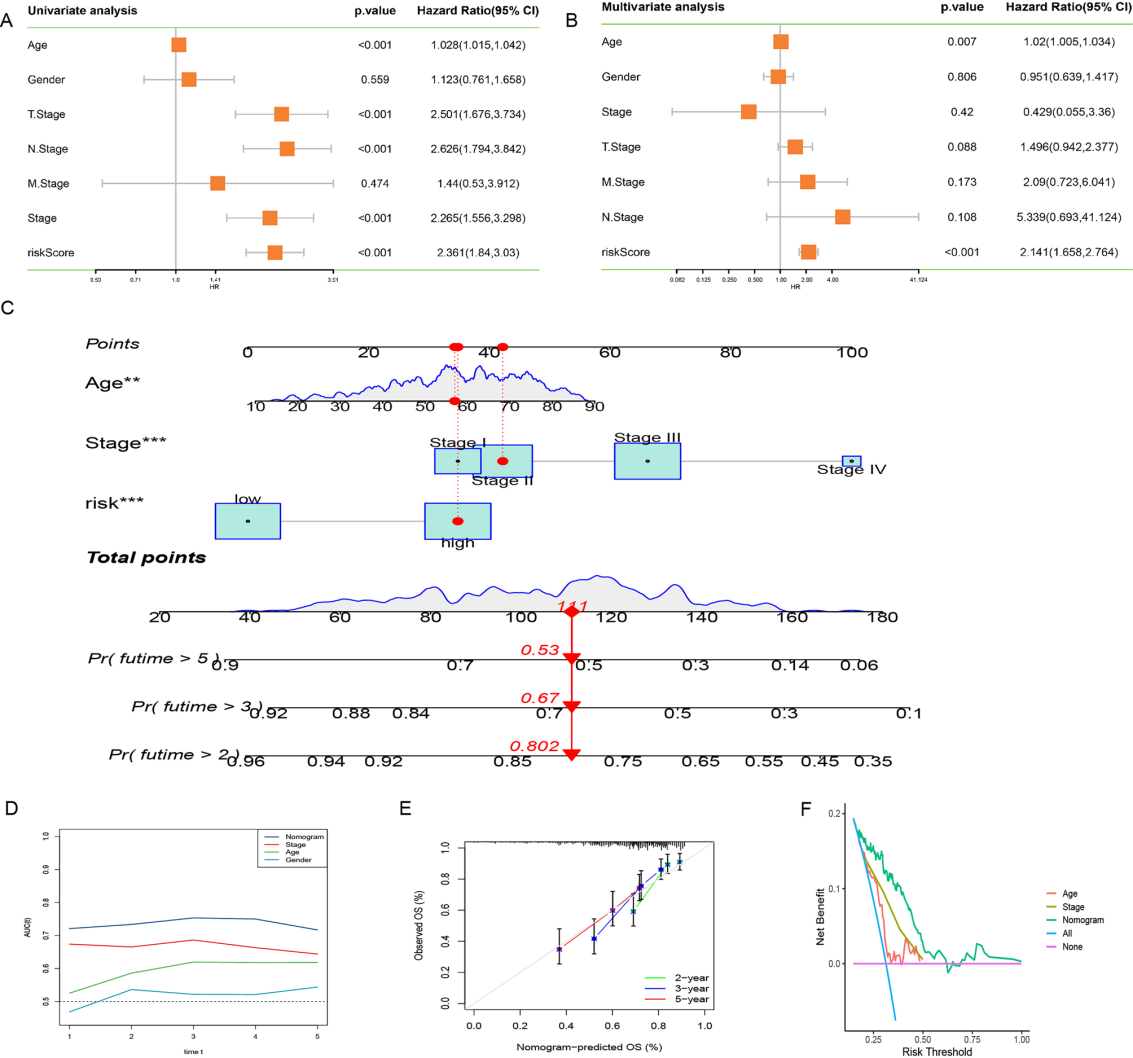
(**A**–**B**) Univariate and multivariate Cox analysis of risk score and clinicopathological features. (**C**) Nomogram integrating risk score and clinical features. (**D**) Comparison of the predictive power of clinicopathologic features and nomogram using timeROC analysis. (**E**) Calibration of the nomogram at 2, 3 and 5 years in the TCGA cohort. (**F**) Decision curve for nomogram.

Given the notable association between the risk score and the advanced and highly malignant nature of SKCM, our inquiry delved into determining whether this risk score could emerge as an independent prognostic factor within the clinical landscape of SKCM. This exploration was undertaken through a comprehensive analysis encompassing both univariate and multivariate Cox regression assessments (Fig. 7A,B). In this analytical context, risk score, age, gender, stage, and TNM staging were all considered as covariates for meticulous evaluation. Intriguingly, the findings underscored the autonomy of age and risk score as independent factors capable of prognosticating the outcome of SKCM patients (Fig. 7C). Driven by this compelling association, we moved forward to construct a clinically relevant prognostic nomogram, capitalizing on the amalgamation of these clinical attributes. This nomogram stands as a robust quantitative tool, effectively poised to predict the trajectory of prognosis, particularly within the context of a worse prognosis for individual patients. The predictive accuracy of the nomogram was methodically verified through the utilization of calibration curves, which aptly gauged the alignment between predicted and observed mortality rates among patients afflicted with measured SKCM. This predictive framework was further strengthened by employing "TimeROC" analysis, which strikingly revealed that the AUC of the nomogram eclipsed the alternative metrics in the TCGA cohort (Fig. 7D). Remarkably, the calibration curves consistently demonstrated the nomogram's propensity to closely mirror actual overall survival, reinforcing its predictive prowess across 2, 3, and 5-year timeframes for melanoma patients (Fig. 7E). Furthermore, DCA curves, enacted across a spectrum of variables within the TCGA cohort, underscored the pronounced benefits conferred by clinical interventions based on the nomogram (Fig. 7F). Notably, patients subjected to therapeutic strategies guided by the nomogram exhibited considerably enhanced rates of favorable outcomes, surpassing the efficacy of interventions solely informed by single clinical characteristics. Collectively, this multifaceted analysis highlights the nomogram's potent utility as a tool that extends beyond prognostic indications, exhibiting a capacity to guide clinical decision-making and optimize therapeutic interventions for melanoma patients.

## Discussion

Cutaneous melanoma, the most prevalent form of skin cancer, has exhibited a rapid surge in incidence over the recent decades. Distinguished by its immunogenicity, melanoma holds promise for favorable responses to immunotherapeutic interventions. Nonetheless, akin to numerous malignancies, melanoma employs a spectrum of inhibitory strategies to circumvent recognition and eradication by the host's innate and adaptive immune mechanisms^49^. The pronounced clinical heterogeneity within the cohort of melanoma patients, coupled with the dearth of early diagnostic markers and therapeutically responsive indicators, underpins the persistently high mortality associated with this disease.

Programmed cell death, or apoptosis, universally ingrained in the tapestry of evolution, assumes a pivotal role in organismal development and the maintenance of homeostasis. This process stands as the cardinal means to eliminate senescent or damaged cells from the body^50^. However, in the backdrop of pathological states, especially in the context of cancer cells, the capacity to orchestrate apoptosis becomes compromised, precipitating unregulated cellular proliferation^51^. Notably, several proteins that experience upregulation in the context of cancer cells have emerged as instrumental in igniting the antiapoptotic cascade response. This has engendered an array of mechanisms facilitating cells' evasion of programmed cell death, including the amplification of anti-apoptotic molecules^52^. The inception of necroptosis as a concept was heralded by Degterev et al. in 2005. In scenarios void of intracellular apoptotic signals, necroptosis engenders an alternative non-apoptotic route to cell demise, typified by necrotic morphology and encompassing elements of autophagy activation^10^. Necroptosis materializes as a caspase-independent mode of cell death, induced solely upon tumor necrosis factor (TNF) exposure when confronted with broad-spectrum cysteine inhibitors such as zVAD fluoromethyl ketone. Unlike apoptosis, the manifestation of necroptosis hinges on the inhibition or perturbation of cysteinyl asparagine 8 function^53^. In the context of cancer therapy, necroptosis emerges as an intriguing alternative to programmed cell death, harnessing the potential to surmount apoptosis resistance while concurrently augmenting anti-tumor immune responses^54^. Research has underscored the pivotal role of necroptosis in governing the migratory and invasive attributes of diverse tumor types^55^. Through its capacity to induce immune activation against tumors, necroptosis emerges as a formidable countermeasure against tumor progression. Existing studies have established the propensity of DNA-damaging chemotherapeutic agents to invoke necroptosis, thereby instigating cancer cell demise via the RIP1/RIP3/MLKL pathway. Noteworthy examples encompass paclitaxel, etoposide, and 5-fluorouracil (5-FU), all of which enact RIP1-induced necroptosis across diverse cancer cell types. In melanoma cells actively engaged in melanogenesis, the use of melanin synthesis inhibitors to induce depigmentation has shown to restore sensitivity to RIPK1/RIPK3/MLKL-mediated necroptosis^56^. Melanin, known for its protective properties and ability to chelate metals, significantly influences the chemosensitivity of melanoma cells towards antitumor drugs^57^. Studies have highlighted that patients exhibiting an unpigmented phenotype in advanced connective tissue-promoting proliferative melanoma exhibit enhanced immunotherapeutic responses to PD-1 or PDL1 immune checkpoint blockade therapies^58^. Intriguingly, necroptosis-induced inflammatory responses have been documented as beneficial in antitumor therapeutic contexts^59,60^. A case in point is the activation of necroptosis through ragweed administration for gastric cancer treatment^61^.

Within our study, a panel of six necroptosis-related genes (GBP4, HSD11B1, CD40, VAMP8, DLL3, and SEMA6A) was initially discerned through the creation of necroptosis prognostic indicators, subsequently forecasting the survival prognoses of SKCM patients within a test set. Moreover, we have constructed a diagnostic model characterized by robust predictive capabilities, employing the risk-associated genes DLL3 and SEMA6A, both of which exhibit a hazard ratio (HR) exceeding 1. Delta-like protein 3 (DLL3), a pivotal ligand in the Notch signaling pathway, is notably abundant in various solid tumors, including melanoma^62^. Substantiated investigations have elucidated that the downregulation of DLL3 can attenuate lipopolysaccharide-induced inflammation, inhibit migration, and impede invasion of melanoma cells through the inhibition of Twist1-mediated epithelial-mesenchymal transition^63^. SEMA6A, a constituent of the semaphorin family, collaborates with plexin to orchestrate crucial aspects of cellular function, including the regulation of the actin cytoskeleton, cellular motility, and proliferation. Recent studies have illuminated SEMA6A's role in governing the viability and proliferation of BRAF^V600E^ human melanoma cells. Additionally, SEMA6A plays a pivotal role in orchestrating the coordinated escape of melanoma cells from concurrent BRAF/MEK inhibition, signifying its potential as a dependable marker for immediate therapeutic benefits of such inhibition. This suggests that SEMA6A could serve as a promising treatment target for BRAF^V600E^ melanoma^64,65^. As evident from the foregoing discussion, DLL3 and SEMA6A emerge as pivotal components in the diagnosis and management of SKCM, offering valuable insights into potential therapeutic interventions. Employing GSVA analysis, we ascertained the marked enrichment of immune-related signaling pathways within NRG cluster A. This encompassed pathways linked to apoptosis, RIG-1-like receptor signaling, and leukocyte transendothelial migration, among others. This underscores the potential for NRGs to orchestrate necroptosis within melanoma, through modulation of intricate immune pathways. We transitioned to stratify patients utilizing a necroptosis risk model, delineating high-risk and low-risk categories that exhibited distinct prognostic outcomes within the two gene clusters. A deep dive into immune infiltration analysis yielded insight into the critical role played by the melanoma tumor microenvironment (TME). Through the prism of GO and KEGG analyses, we gleaned that NRGs could exert influence over the composition of the tumor immune microenvironment, consequently shaping SKCM's developmental trajectory. The TME, characterized by a blend of natural immune cells (such as macrophages, mast cells, neutrophils, and dendritic cells) and acquired immune cells (T and B lymphocytes), interacts with tumor cells via diverse avenues—direct contact or cytokine signaling—culminating in a profound influence over tumor behavior and therapeutic responsiveness^66^. Strikingly, the diminished immune infiltration abundance observed in the high-risk melanoma group alludes to an overall impairment in immune function. This conjecture is fortified by parallel immunotherapy response analyses, which laid bare shorter overall survival and inferior immunotherapy response for high-risk melanoma patients.

Given its status as a paradigmatic immunogenic tumor, the correlation between immune cell infiltration and melanoma prognosis is contingent upon the nature and presence of immune cells within the tumor TME. In the context of our study, we meticulously evaluated the connection between the necroptosis risk model and immune cell infiltration abundance. The findings revealed a significant correlation between most immune cells and the risk score. Notably, the risk score displayed a positive correlation with M0 macrophages while manifesting negative correlations with M1 macrophages, CD4^+^ T cells, and CD8^+^ T cells. This resonated within the low-risk group, characterized by heightened infiltration levels of CD8^+^ T cells, CD4^+^ T cells, and M1 macrophages. It is widely established that M1 macrophages enhance melanoma patient prognosis under their roles in inflammation promotion and tumor suppression, underpinned by the secretion of proinflammatory cytokines like IL-12 and TNF-α, alongside robust expression of nitric oxide synthase (iNOS)^67,68^. The prominence of CD8^+^ T cells also underscores their pivotal role in orchestrating antitumor immunity, thereby correlating with prolonged overall survival. In contrast, M0 macrophages, predominant in the high-risk group, suggest a close nexus with melanoma progression and metastasis. This conjecture finds resonance with studies that highlight the heightened prevalence of M0 macrophages in the N1 stage of colorectal cancer, attesting to their correlation with tumor metastasis^69^. It is worth noting that Treg cells exhibited higher proportions within the high-risk cohort compared to the low-risk cohort. This disparity could potentially be attributed to the role of Treg cells in regulating oxidative stress and the hyperactive inflammatory responses that necroptosis engenders within the TME. In the context of colon cancer, these Treg cell subtypes have demonstrated opposing functions in modulating the TME^70^.

Taking into account the far-reaching influence of necroptosis on melanoma's heterogeneous nature and its corresponding clinical ramifications, we devised a necroptosis risk model hinging on six risk-associated genes, accompanied by a quantified risk score nomogram. These compelling findings substantiate the utility of the necroptosis risk model score as an autonomous prognostic biomarker for melanoma patients. Our proactive foray into antitumor drug sensitivity analyses across disparate risk subgroups has equipped the necroptosis risk model with the prowess to prognosticate and identify promising drug candidates.

Yet, it is imperative to acknowledge certain limitations inherent to our study. Foremost, although we have successfully identified some necroptosis-related prognostic gene markers pertinent to SKCM, further validation through in vitro and in vivo experiments is imperative for a more comprehensive understanding. Secondly, considering that the clinical cohorts under scrutiny are predominantly of Caucasian ethnicity, a broader validation encompassing diverse ethnic cohorts becomes indispensable. In conclusion, the imperative to undertake multicenter clinical cohorts for exhaustive validation underscores the next critical juncture in advancing our findings.

## Conclusion

In summation, our study undertook a meticulous analysis of necroptosis-related genes, encompassing their multifaceted influence on the immune microenvironment, clinicopathological attributes, and overall prognosis. The culmination of our efforts resulted in the establishment of a robust necroptosis risk model, complemented by an insightful antitumor drug sensitivity assessment. This collective endeavor not only augments the repertoire of potential biomarkers but also holds promise for enhancing the prognostic and therapeutic landscape of patients afflicted with clinical SKCM. The implications of our findings extend to the realm of personalized treatment strategies, poised to address the distinct melanoma subtypes exhibited by diverse patients.

## Data and code availability

The datasets generated and analyzed during the current study can be found in the TCGA(https://portal.gdc.cancer.gov/repository), GEO database(http://www.ncbi.nlm.nih.gov/geo/), including GSE54467,GSE53118,GSE65904,GSE19234,GSE78220, and GSE15605, and UCSC repository (https://xenabrowser.net/datapages/). The corresponding author will provide the code used in this work upon reasonable request.

### Supplementary Information

Supplementary Information 1.

Supplementary Information 2.

Supplementary Information 3.
