# Supplementary material for: Comprehensive analysis of necroptotic patterns and associated immune landscapes in individualized treatment of skin cutaneous melanoma
Source: Sci Rep. 2023 Nov 30;13:21094. doi: 10.1038/s41598-023-48374-0 (PMC10689831; doi:10.1038/s41598-023-48374-0)
Supplement: Supplementary file 1 — Supplementary Information 1. [file 41598_2023_48374_MOESM1_ESM.docx]

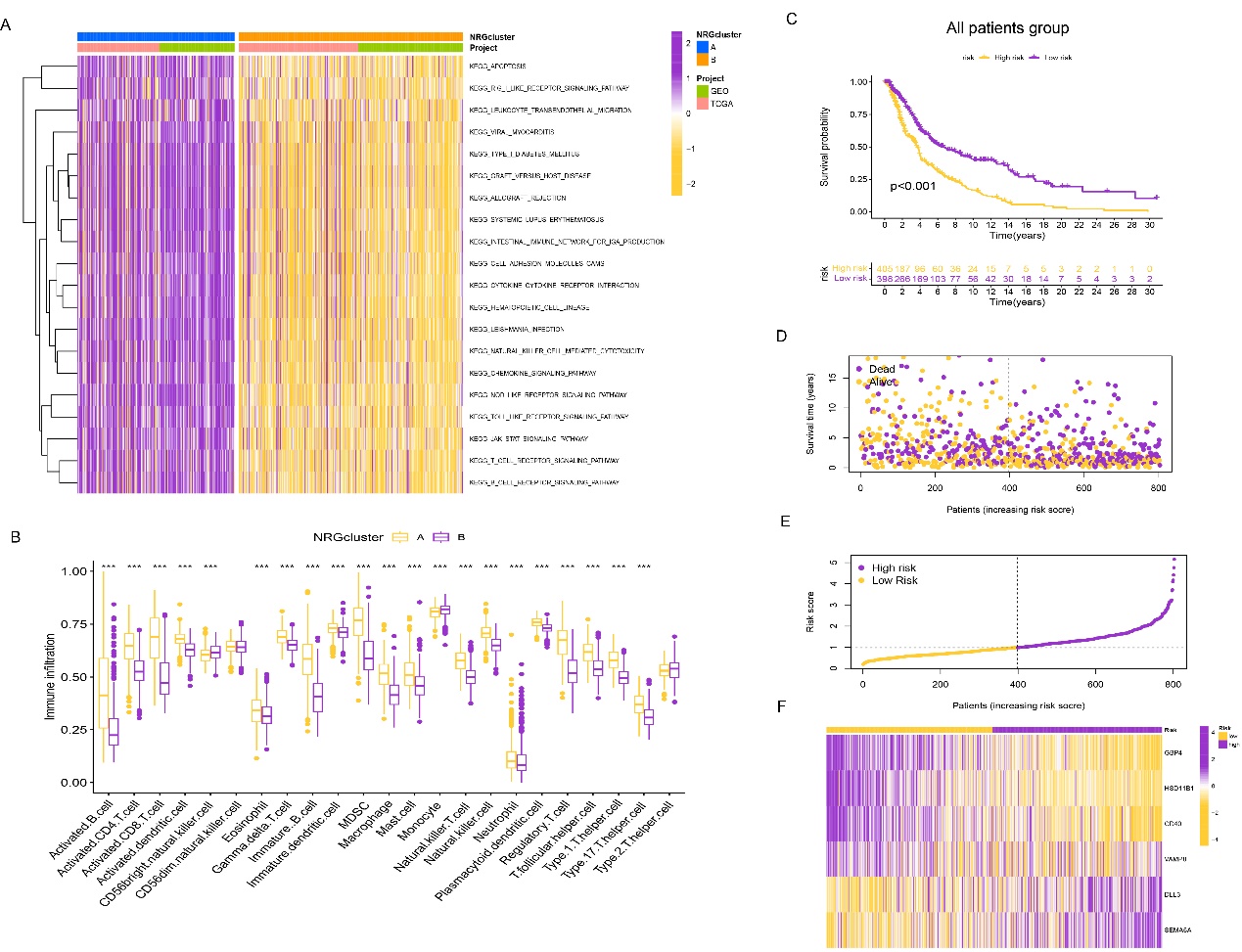


Supplementary Figure S1 **A** GSVA of two different kinds of biological pathways, where purple and yellow correspondingly represent activated and inhibited pathways. **B** The prevalence of 23 immune cell types that infiltrate in the two SKCM subgroups. **C** Over survival Kaplan-Meier curves for the entire patient group. **D-F** The survival status and risk score distribution throughout the entire patient population.


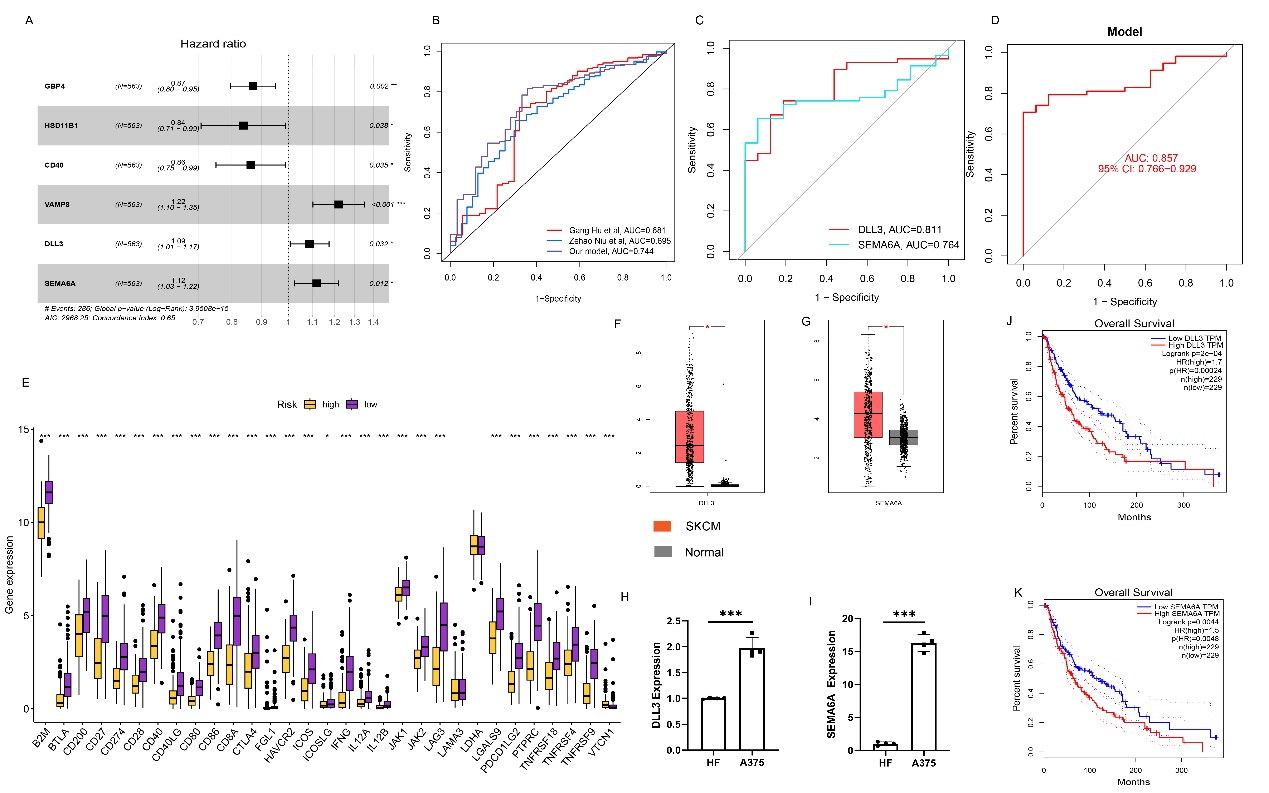


Supplementary Figure S2 **A** The six genes that make up the risk scoring model. **B** Comparison of our model with other necroptotic prognostic models. **C-D** ROC curves for diagnostic model constructed by DLL3 and SEMA6A. **E** Differential expression of immune checkpoints in different risk subgroups. **F-G** Exploring the expression patterns of DLL3 and SAMA6A through the GEPIA Database. **H-I** The expression of risk genes in A375(SKCM) cell and human fibroblasts (HF) was validated by RT-qPCR. **J-K** Association of DLL3 and SAMA6A with prognosis obtained from the GEPIA database.


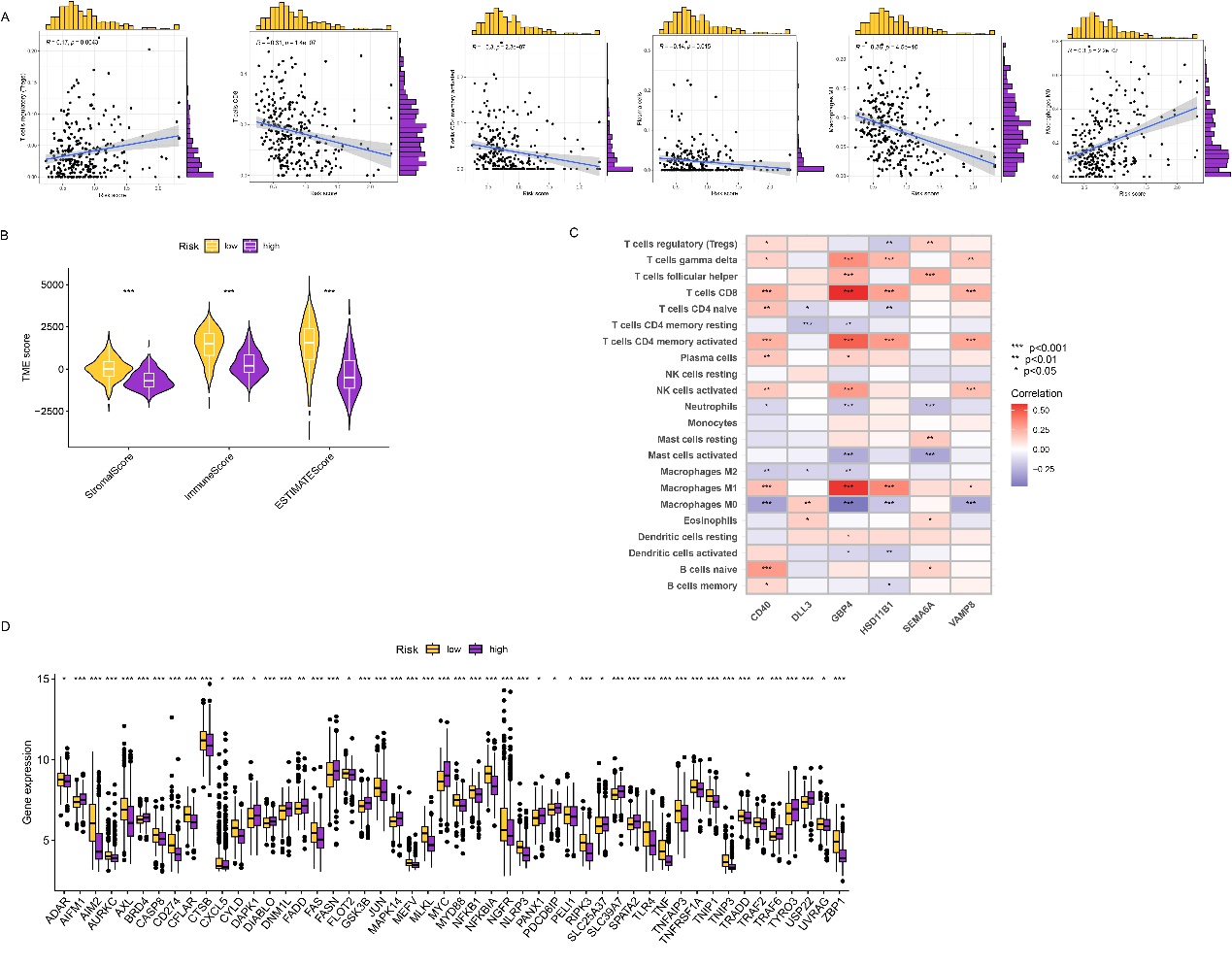


Supplementary Figure S3 **A** Correlations between risk score and immune cell types. **B** Differential expression of stromal, ESTIMATE, and immune scores across risk subgroups. **C** Correlations between the abundance of immune cells and six genes in the proposed model. **D** Expression of necroptosis-related genes in the high- and low-risk groups.
