# Supplementary material for: Comprehensive analysis of necroptotic patterns and associated immune landscapes in individualized treatment of skin cutaneous melanoma
Source: Sci Rep. 2023 Nov 30;13:21094. doi: 10.1038/s41598-023-48374-0 (PMC10689831; doi:10.1038/s41598-023-48374-0)
Supplement: Supplementary file 3 — Supplementary Information 3. [file 41598_2023_48374_MOESM3_ESM.docx]

|  | **High Risk (N=227)** | **Low Risk (N=223)** | **P-value** |
| --- | --- | --- | --- |
| **Age** |  |  |  |
| age <60 | 109 (48.0%) | 131 (58.7%) | 0.0288 |
| age >= 60 | 118 (52.0%) | 92 (41.3%) |  |
| **Gender** |  |  |  |
| female | 77 (33.9%) | 91 (40.8%) | 0.158 |
| male | 150 (66.1%) | 132 (59.2%) |  |
| **Stage** |  |  |  |
| i/ii nos | 3 (1.3%) | 7 (3.1%) | 0.00598 |
| not reported | 13 (5.7%) | 20 (9.0%) |  |
| stage 0 | 3 (1.3%) | 3 (1.3%) |  |
| stage i | 29 (12.8%) | 48 (21.5%) |  |
| stage ii | 85 (37.4%) | 48 (21.5%) |  |
| stage iii | 84 (37.0%) | 86 (38.6%) |  |
| stage iv | 10 (4.4%) | 11 (4.9%) |  |
| **Event** |  |  |  |
| Yes | 129 (56.8%) | 93 (41.7%) | 0.00184 |
| No | 98 (43.2%) | 130 (58.3%) |  |
| **M** |  |  |  |
|  | 12 (5.3%) | 12 (5.4%) | 0.889 |
| M0 | 205 (90.3%) | 199 (89.2%) |  |
| M1 | 10 (4.4%) | 12 (5.4%) |  |
| **N** |  |  |  |
|  | 6 (2.6%) | 11 (4.9%) | 0.83 |
| N0 | 116 (51.1%) | 107 (48.0%) |  |
| N1 | 35 (15.4%) | 38 (17.0%) |  |
| N2 | 24 (10.6%) | 25 (11.2%) |  |
| N3 | 29 (12.8%) | 27 (12.1%) |  |
| NX | 17 (7.5%) | 15 (6.7%) |  |
| **T** |  |  |  |
|  | 9 (4.0%) | 16 (7.2%) | <0.001 |
| T0 | 5 (2.2%) | 18 (8.1%) |  |
| T1 | 15 (6.6%) | 26 (11.7%) |  |
| T2 | 32 (14.1%) | 45 (20.2%) |  |
| T3 | 44 (19.4%) | 46 (20.6%) |  |
| T4 | 97 (42.7%) | 48 (21.5%) |  |
| Tis | 4 (1.8%) | 3 (1.3%) |  |
| TX | 21 (9.3%) | 21 (9.4%) |  |
| **Breslow_depth** |  |  |  |
| I(≤0.75mm） | 8 (3.5%) | 25 (11.2%) | <0.001 |
| II(0.76～1.5mm) | 28 (12.3%) | 37 (16.6%) |  |
| III(＞1.5mm) | 147 (64.8%) | 105 (47.1%) |  |
| NA | 44 (19.4%) | 56 (25.1%) |  |
| **Clark_level** |  |  |  |
|  | 65 (28.6%) | 73 (32.7%) | <0.001 |
| I | 3 (1.3%) | 2 (0.9%) |  |
| II | 6 (2.6%) | 12 (5.4%) |  |
| III | 28 (12.3%) | 48 (21.5%) |  |
| IV | 87 (38.3%) | 75 (33.6%) |  |
| V | 38 (16.7%) | 13 (5.8%) |  |
| **Type** |  |  |  |
| Metastatic | 155 (68.3%) | 200 (89.7%) | <0.001 |
| Primary Tumor | 72 (31.7%) | 23 (10.3%) |  |

Table S7 Underlying clinicopathologic features of melanoma patients in TCGA based on high- and low-risk groups.

Gene Symbol Forward Reverse

DLL3 5’- CACTCCCGGATGCACTCAAC-3’ 3’- GATTCCAATCTACGGACGAGC-5’

SEMA6A 5’-AATCAGTATTTCGCATGGCAACT-3’ 3’- GCAATGTAGAGGGTTCCGTTCA-5’

Actin 5’- GTAACCCGTTGAACCCCATT-3’ 3’ - CCATCCAATCGGTAGTAGCG-5’

Table S8 The premier sequences.
